# Supplementary material for: Genome-wide association study reveals multiple loci for nociception and opioid consumption behaviors associated with heroin vulnerability in outbred rats
Source: Mol Psychiatry. 2025 Feb 25;30(8):3363–75. doi: 10.1038/s41380-025-02922-4 (PMC12240846; doi:10.1038/s41380-025-02922-4)
Supplement: Supplementary file 1 — Supplemental materials [file 41380_2025_2922_MOESM1_ESM.docx]

**Supplementary Information**

*Behavioral testing*

Elevated-plus maze (EPM): Sessions were conducted in an elevated apparatus (San Diego Instruments; 4 arms at 110.5 cm long and 10.2 cm wide) that consisted of two “closed” arms (30.5 cm high walls) and two “open” arms (i.e., not enclosed). Animals were placed in the center of the maze to start, and movement was tracked automatically using ANY-maze behavioral tracking software (Stoelting, Wood Dale; version 6.17) over the course of the 5-min session. A minimum of 85% of the rat’s body had to be within an arm for it to be counted. The percent time the animal spent in each arm was calculated.

Open field test (OFT): Animals were placed in the center of a Plexiglas chamber housed within a metal frame (Omnitech Electronics, Columbus, OH; 40.6 cm L x 40.6 cm W x 30.5 cm H) containing photocells that tracked vertical and horizontal movements. Sessions lasted for 60-min and data was recorded and analyzed for distance and time spent moving using Versamax (Omnitech Electronics, Columbus, OH; version 1.80-0142).

Tail flick test (TF): Testing occurred on a platform with the rat’s tail over an infrared light beam equipped with a motion sensor that turned the beam off once the tail was removed, or after 10 seconds (Ugo Basile S.R.L., Gemonio, Italy). TF was comprised of two phases: baseline (1 mg/kg saline injection, s.c.) and test (0.75 mg/kg heroin, s.c.). Injections were administered 15 min prior to testing and the phases were separated by 1 hour. Each test consisted of 4 trials with the location of the tail tested being adjusted by 1 cm each trial to prevent tissue damage. Average latency to remove the tail from the beam was calculated.

*Heroin taking, refraining and seeking measures*

Prior to training, rats were outfitted with an indwelling jugular catheter and post-operatively administered an antibiotic (Cefazolin, 0.2 mg/kg, s.c.; or enrofloxacin, 1 mg/kg, i.v.) and analgesic (Ketorolac, 2 mg/kg, s.c.; or Meloxicam, 0.5 mg/rat, s.c.). Training occurred in standard behavioral testing chambers (Med Associates, St. Albans, VT) housed within a sound attenuating box outfitted with a ventilation fan. Two levers with a light above each were on one chamber wall, and opposite was a house light and speaker. The house light turned on at the start of all sessions. A fixed ratio 1 schedule of reinforcement was used for heroin self-administration whereupon a press on the active lever resulted in an infusion of heroin (20 µg/kg/100 µl infusion over 3-sec) and a 5-sec presentation of a light/tone cue. The house light turned off for 20-sec at the time of infusion to signal a time out period during which additional active lever presses were recorded but without consequence. Four training sessions (12-h or 300 infusions earned) occurred per week (Monday-Friday) with one randomized day off per week, totaling 12 sessions. The progressive ratio test occurred next to assess heroin break point. Testing terminated after 12-h or 1-h of no earned infusion. Heroin self-administration training was then re-established for 3 days, followed by a within-session extinction-prime test lasting 6 hours. Testing occurred under extinction conditions (i.e., active lever presses no longer resulted in heroin infusion or light/tone cue presentation). With two hours left in the session, animals received a heroin prime injection (0.25 mg/kg, s.c.). Extinction training session and a test for cued reinstatement followed. Throughout testing, presses on the inactive lever were recorded but had no consequence.

*GWAS analysis*

A detailed report of all GWAS findings may be found in the included ZIP file.
