# Supplementary material for: Genome-wide association study reveals multiple loci for nociception and opioid consumption behaviors associated with heroin vulnerability in outbred rats
Source: Mol Psychiatry. 2025 Feb 25;30(8):3363–75. doi: 10.1038/s41380-025-02922-4 (PMC12240846; doi:10.1038/s41380-025-02922-4)
Supplement: Supplementary file 2 — Supplemental tables and figures [file 41380_2025_2922_MOESM2_ESM.docx]

**
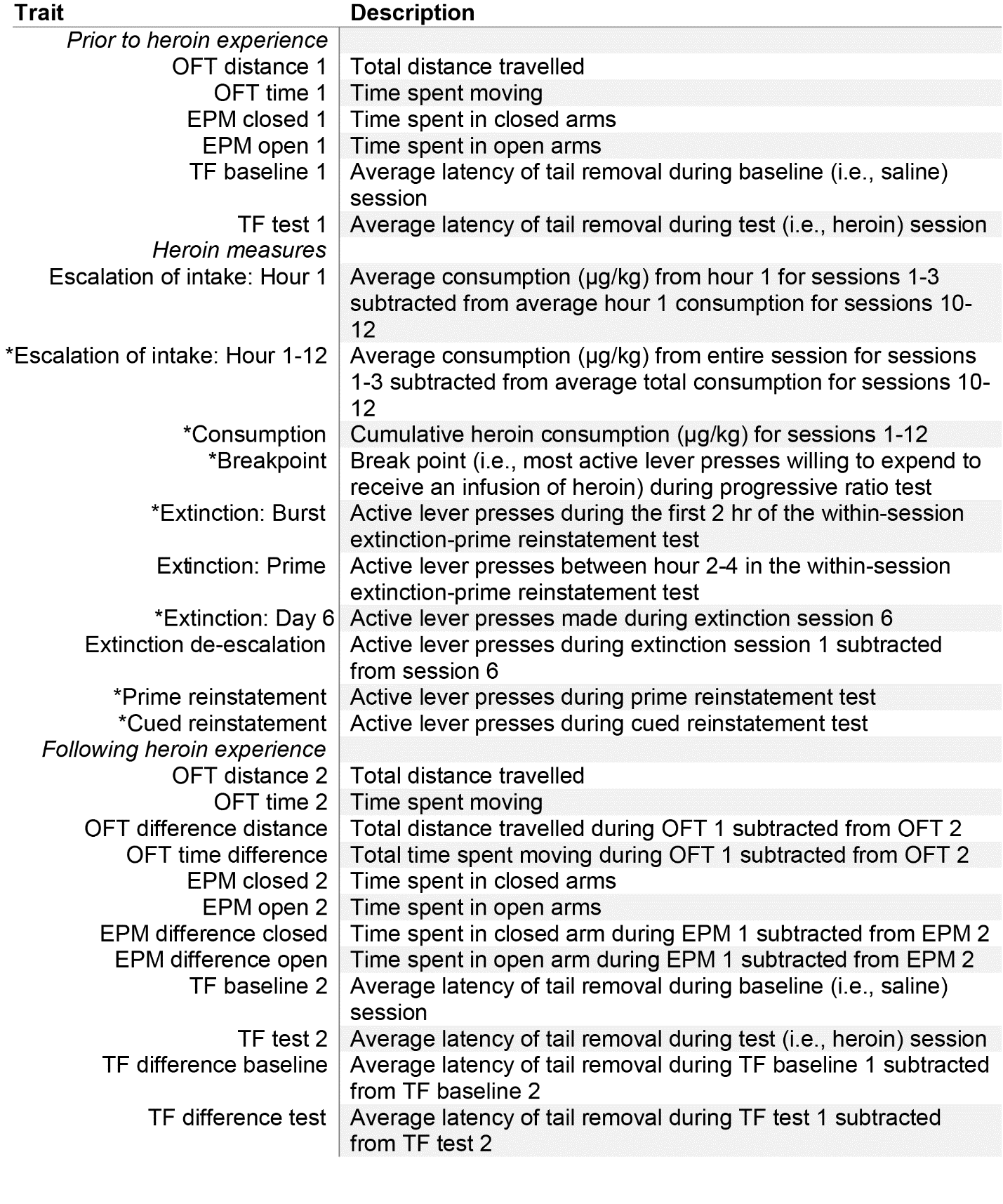
Supplemental Table 1** Description of behavioral traits assessed in GWAS. Traits included those measured both prior to (time point 1) and following (time point 2) heroin experience, and the difference between the two time points, along with several behaviors associated with heroin taking, refraining and seeking. Traits used in the non-linear clustering approach to categorize rats into OUD phenotypes are designated with an asterisk.


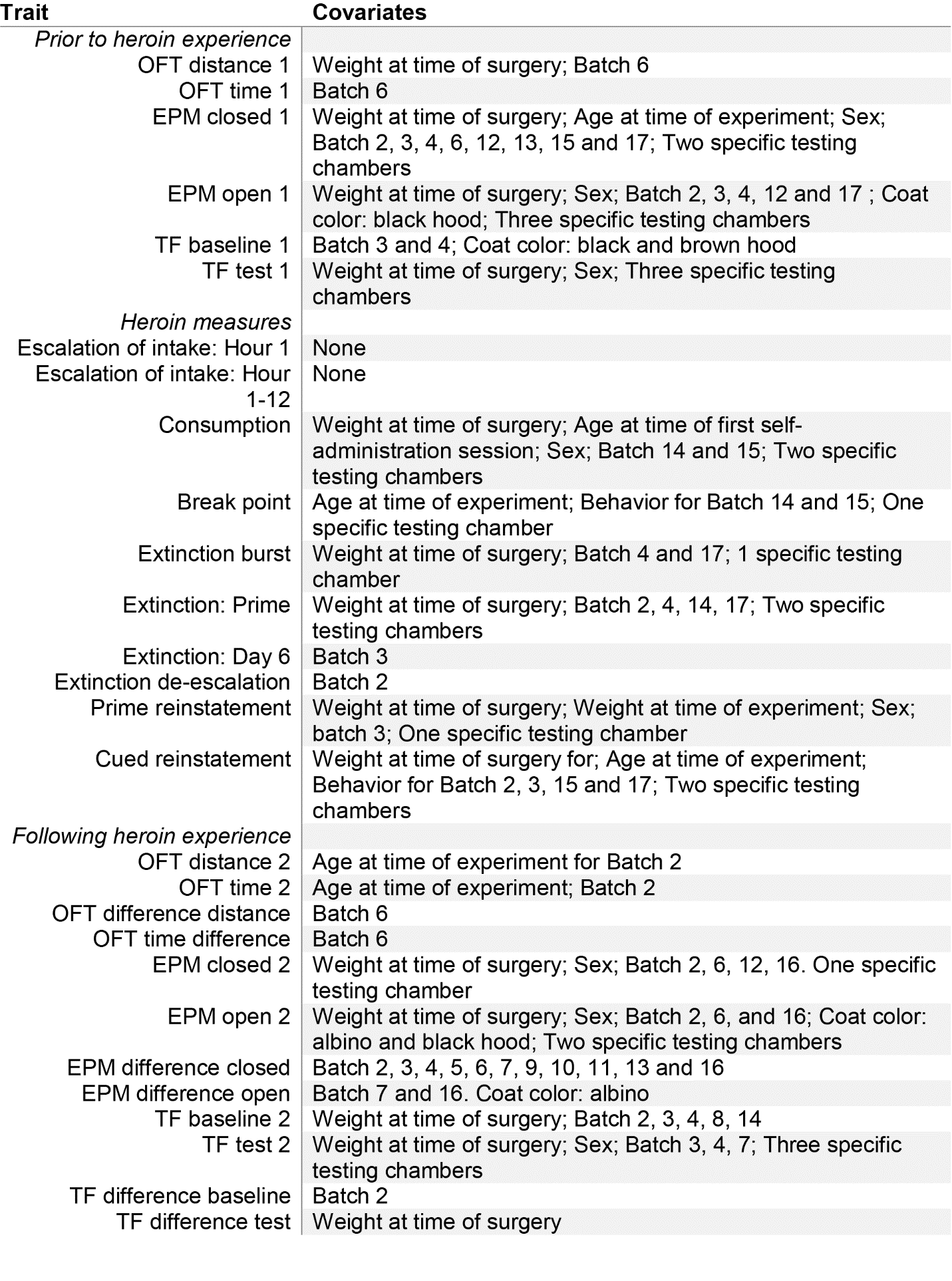


**Supplemental Table 2** Covariates for behavioral traits from rats tested at MUSC. Covariates that explained >2% of variance were regressed out prior to further analyses. Batches of 40 rats (20 males and 20 females) were shipped every few months from early 2019 through the end of 2022. Testing chamber refers to behavioral testing chamber that all heroin taking, extinction and seeking occurred in. Heterogeneous rats have 5 distinct coat colors: albino, black, brown, black hood and brown hood.


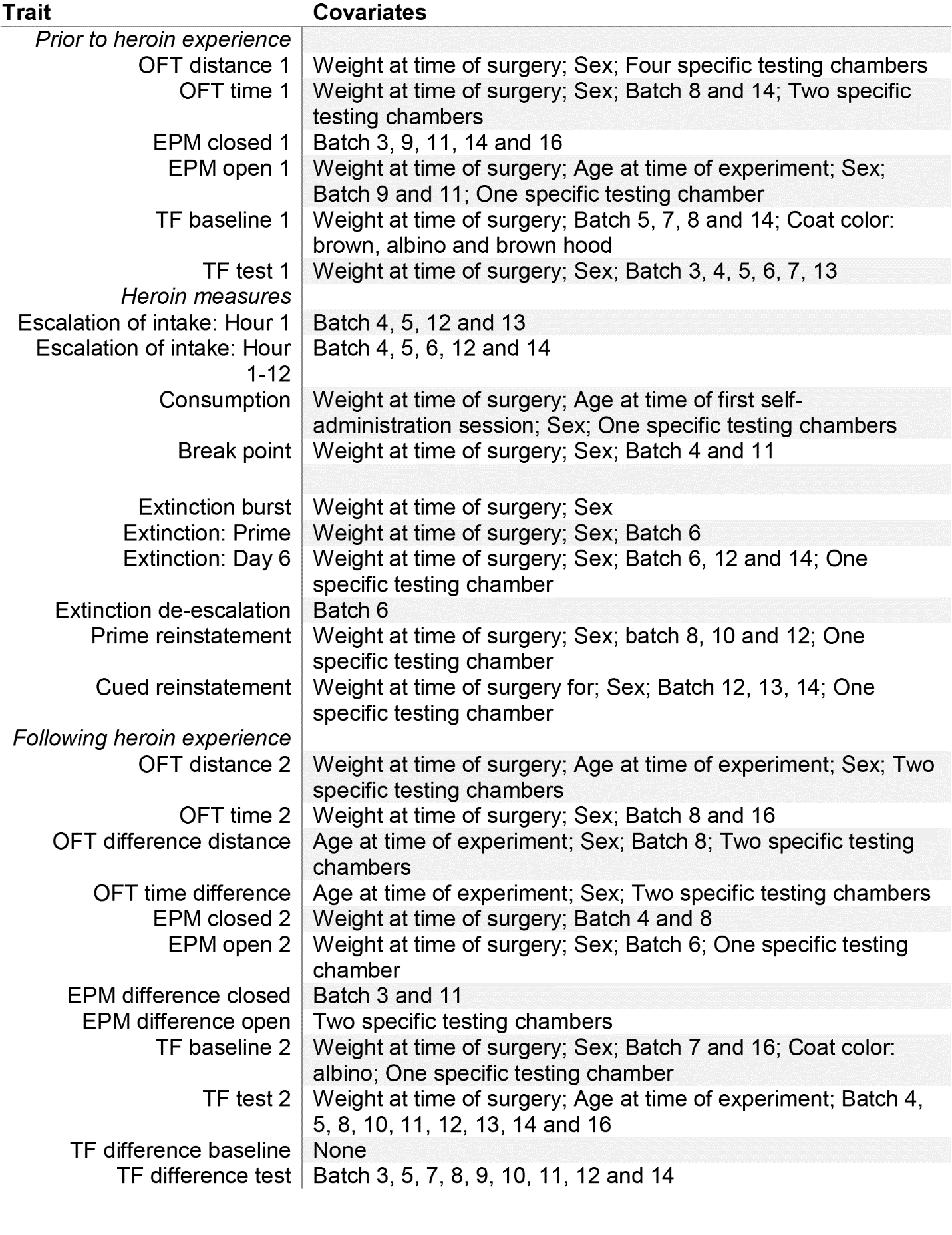


**Supplemental Table 3** Covariates for behavioral traits from rats tested at UCAM. All covariates that explained >2% of variance for a trait were regressed out prior to continuing analyses. Rats were shipped every few months from early 2019 through the end of 2022 in batches of 40 rats (20 males and 20 females). Testing chamber refers to behavioral testing chamber that all heroin taking, extinction and seeking occurred in. Heterogeneous rats have 5 distinct coat colors: albino, black, brown, black hood and brown hood.


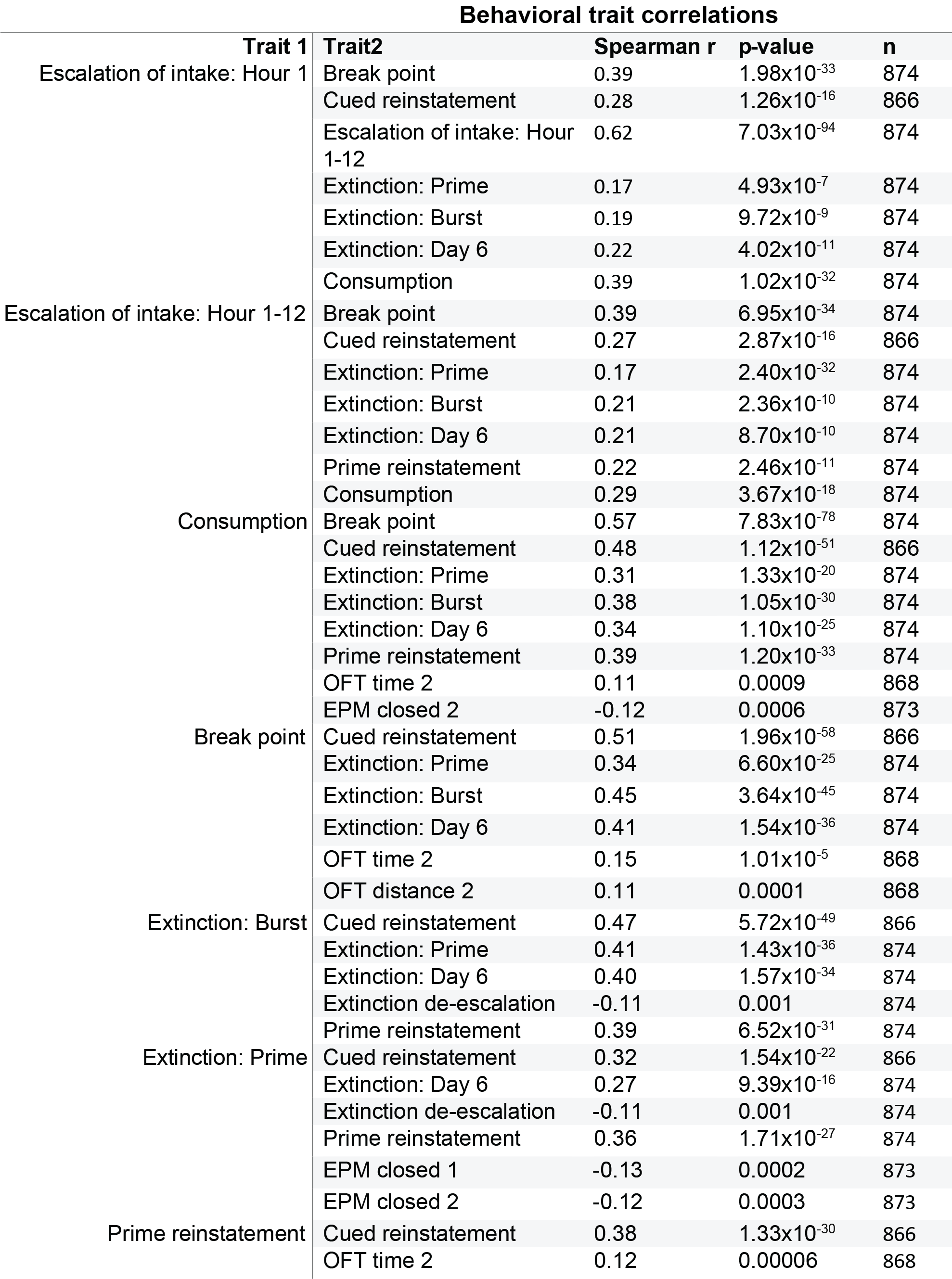


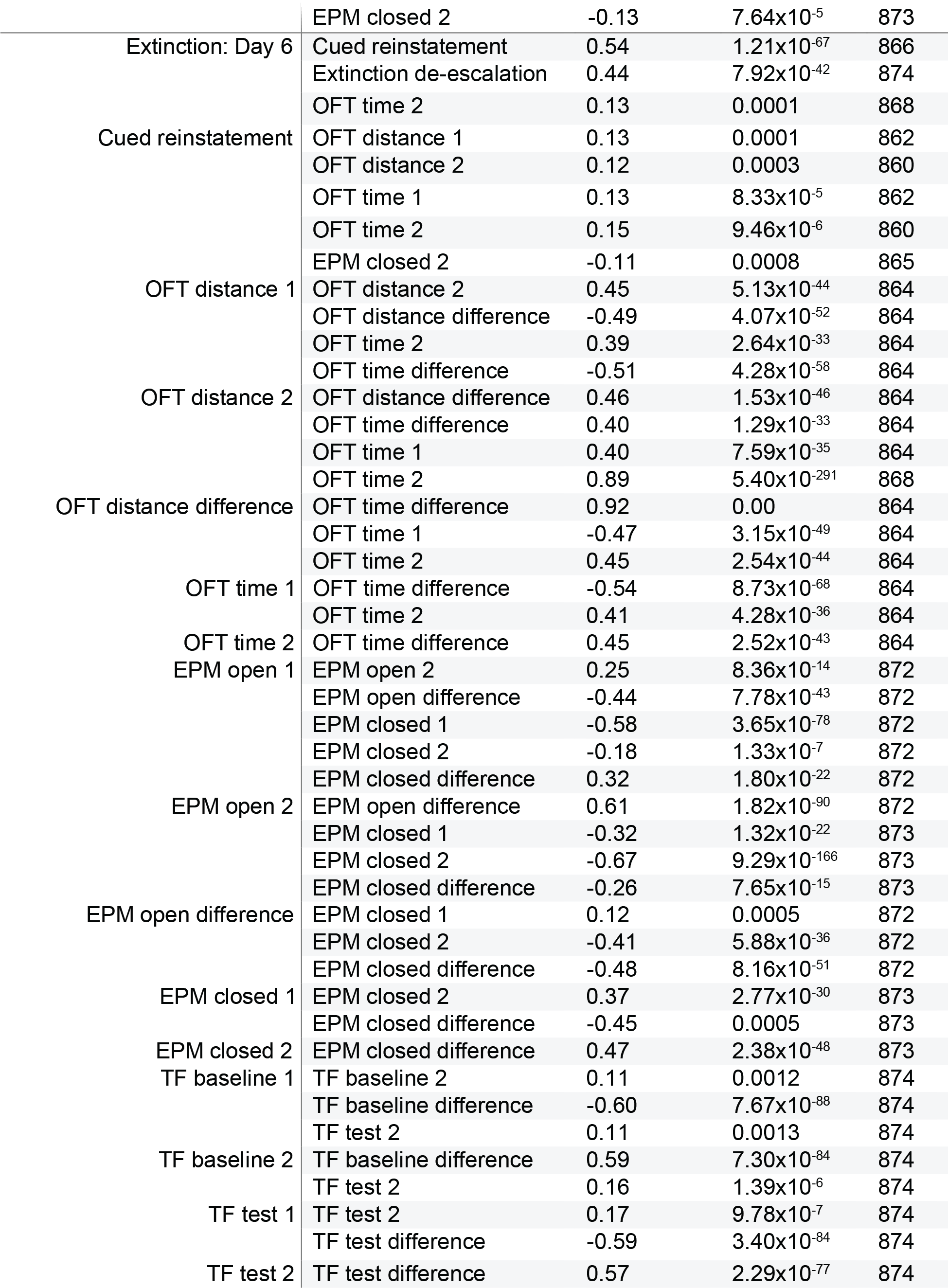


**Supplemental Table 4** Data statistics for significant correlated behavioral traits. Heroin taking, refraining and seeking behaviors showed the tendency to co-vary with one another, whereas behaviors assessing stress- and anxiety-like behaviors as well as nociceptive threshold exhibited greater within-test covariance. Direction and magnitude of correlation (Spearman r) and total number of animals included in analysis (n) are included in the table. A significance threshold of p<0.05 with Bonferroni adjustment for correction for multiple comparisons was implemented, resulting in a p<0.0018 in order to attain significance.


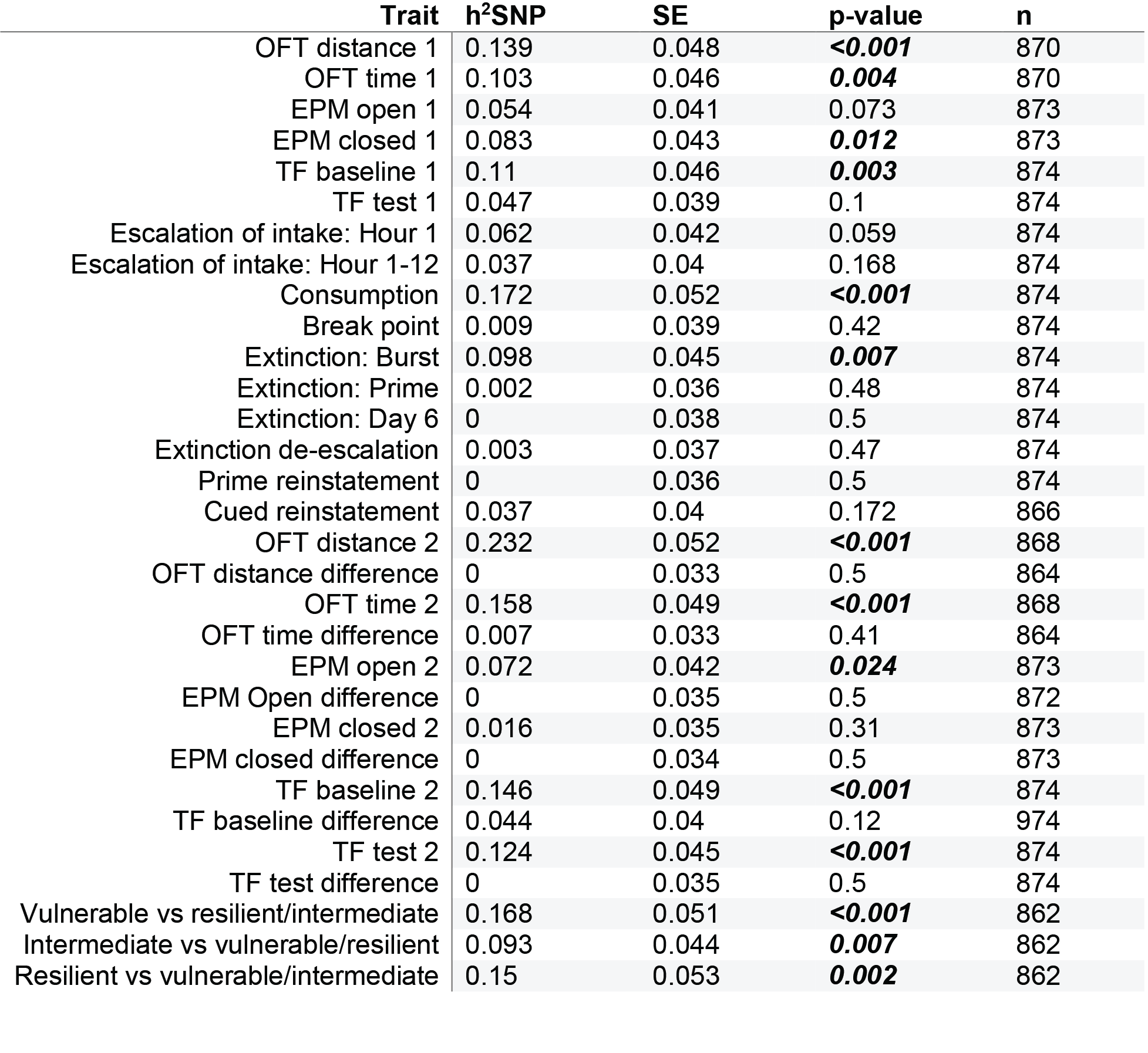
**Supplemental Table 5** SNP heritability table for behavioral traits. Significant relationships are highlighted in bold. The heritability index (h^2^SNP), standard error (SE), p-value and total number of animals in analysis (n) for each trait is recorded.

**
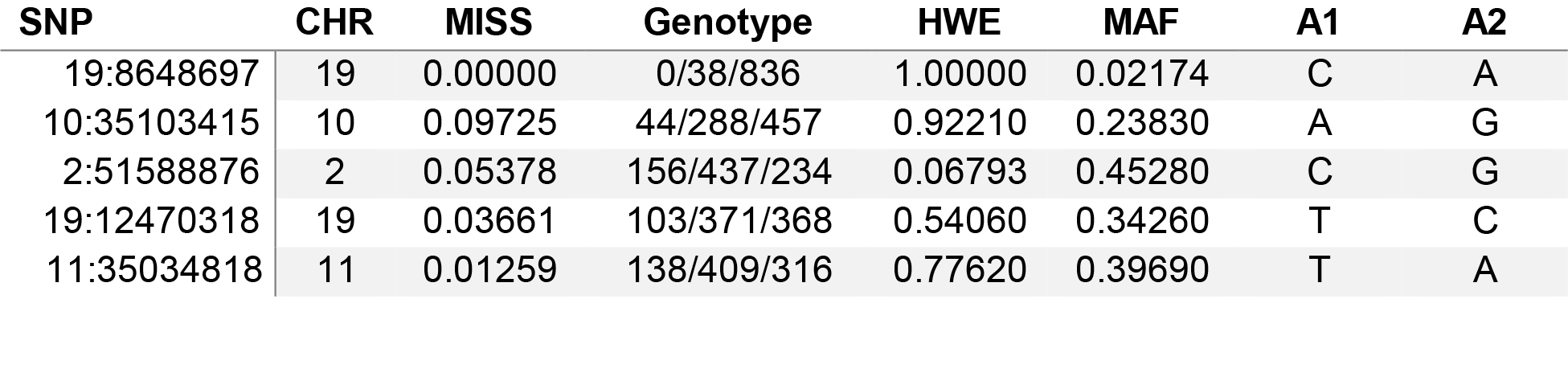
**

**Supplemental Table 6** Minor allele frequency and Hardy-Weinberg equilibrium values for significant QTLs. For each SNP identified, the chromosome number (CHR), missingness (MISS), genotype (count of reference/reference, reference/alternate, and alternate/alternate alleles), p-value of Hardy-Weinberg equilibrium (HWE; assumes H=0 is in HWE), minor allele frequency (MAF), non-reference allele (A1) and reference allele (A2). All SNPs passed the filters for MISS, HWE and MAF.


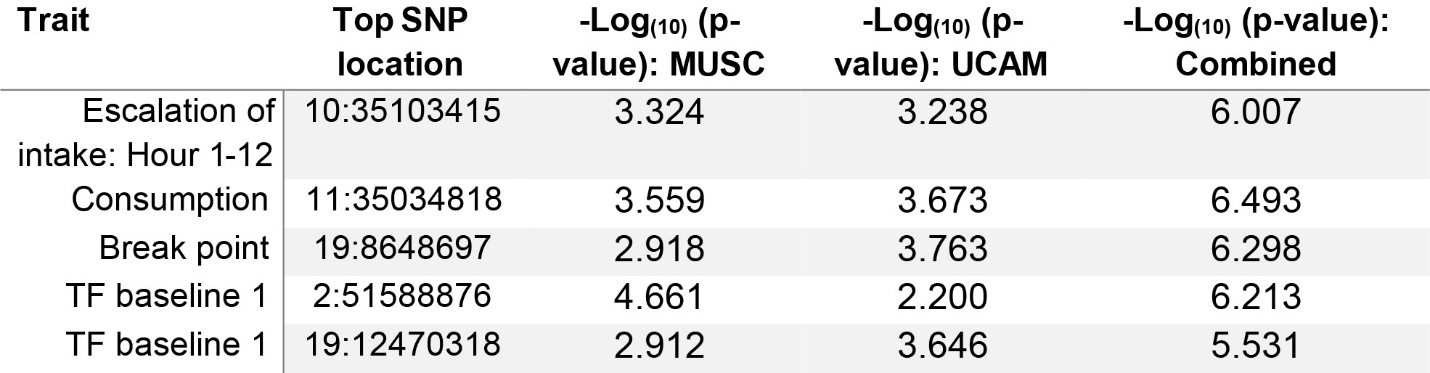


**Supplemental Table 7** Comparison of GWAS results between sites. Table shows significant QTLs conserved between GWAS analysis when combining sites versus considering each side separately. Each trait, p-value (-log_10_) and chromosome location (chromosome: location) is recorded for each significant QTL. (MUSC, n=479; UCAM, n=395)


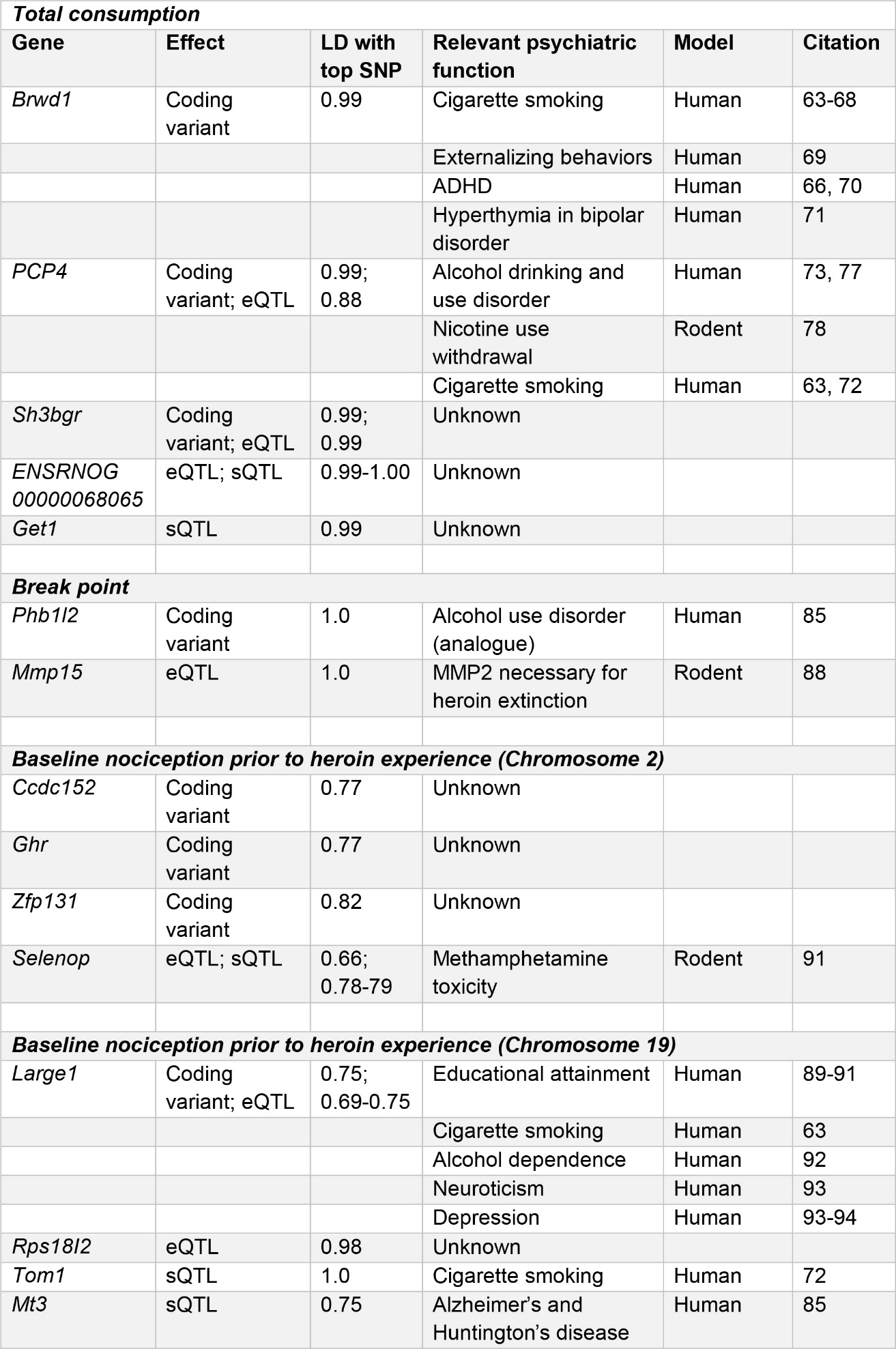


**Supplemental Table 8** Summarization of significant QTLs for selected traits and relevance to psychiatric disorders. The gene, effect (relationship to peak SNP), linkage disequilibrium (LD) to peak SNP, known functional role in psychiatric illnesses, model organisms used and citation for the study are recorded.


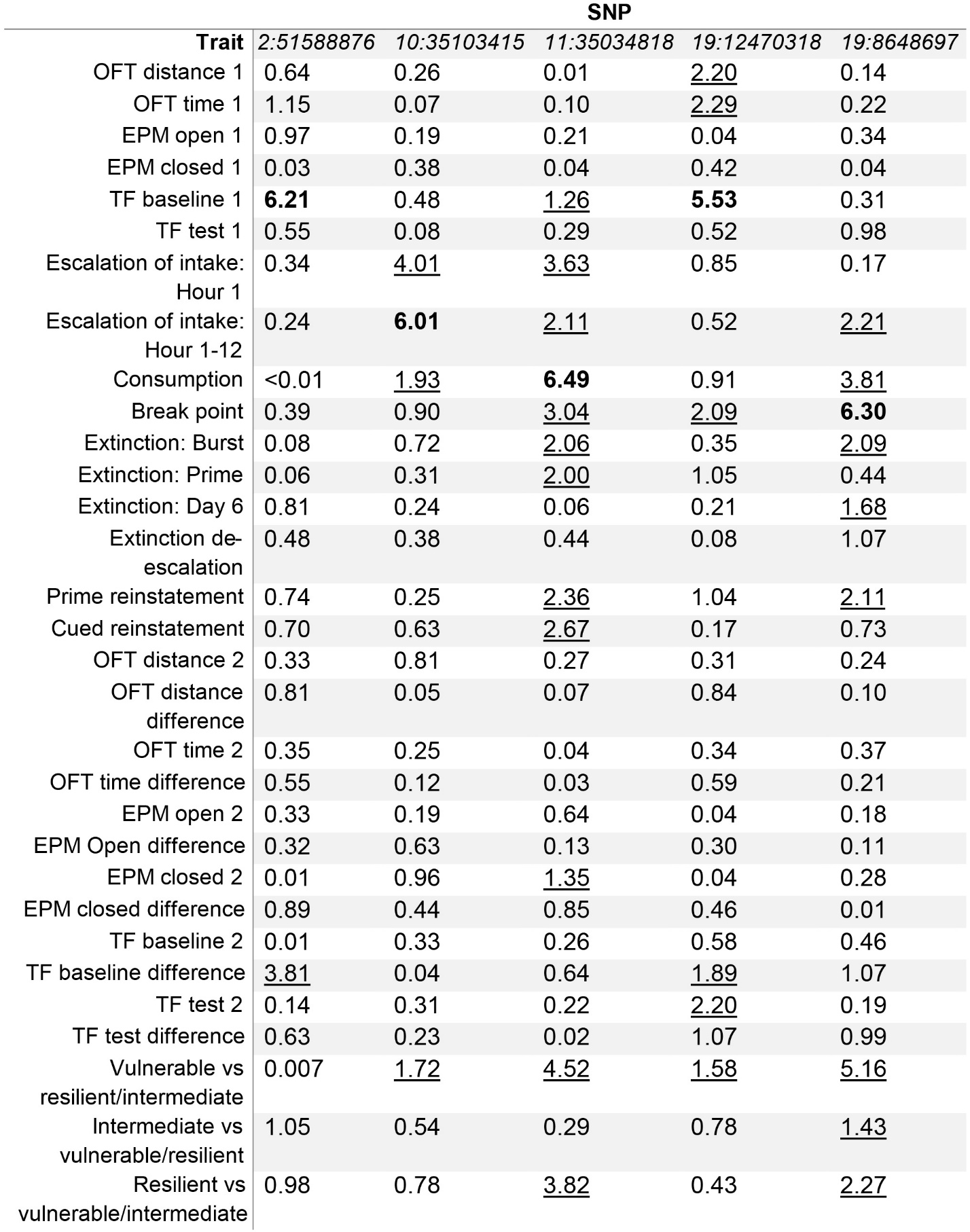


**Supplemental Table 9** Significant SNPs across all behavioral traits tested. The p-value (-log_10_) for the significant SNPs identified in the GWAS analysis across all behavioral traits. Values in bold indicate genome-wide significant p-values; underlines indicate p-values greater than -log(p) = 1.3 (-log(p) of 1.3 = 0.05) but below the threshold for genome-wide significance of -log(p)=5.58 (-log(p) = 5.58 = 0.00000263)


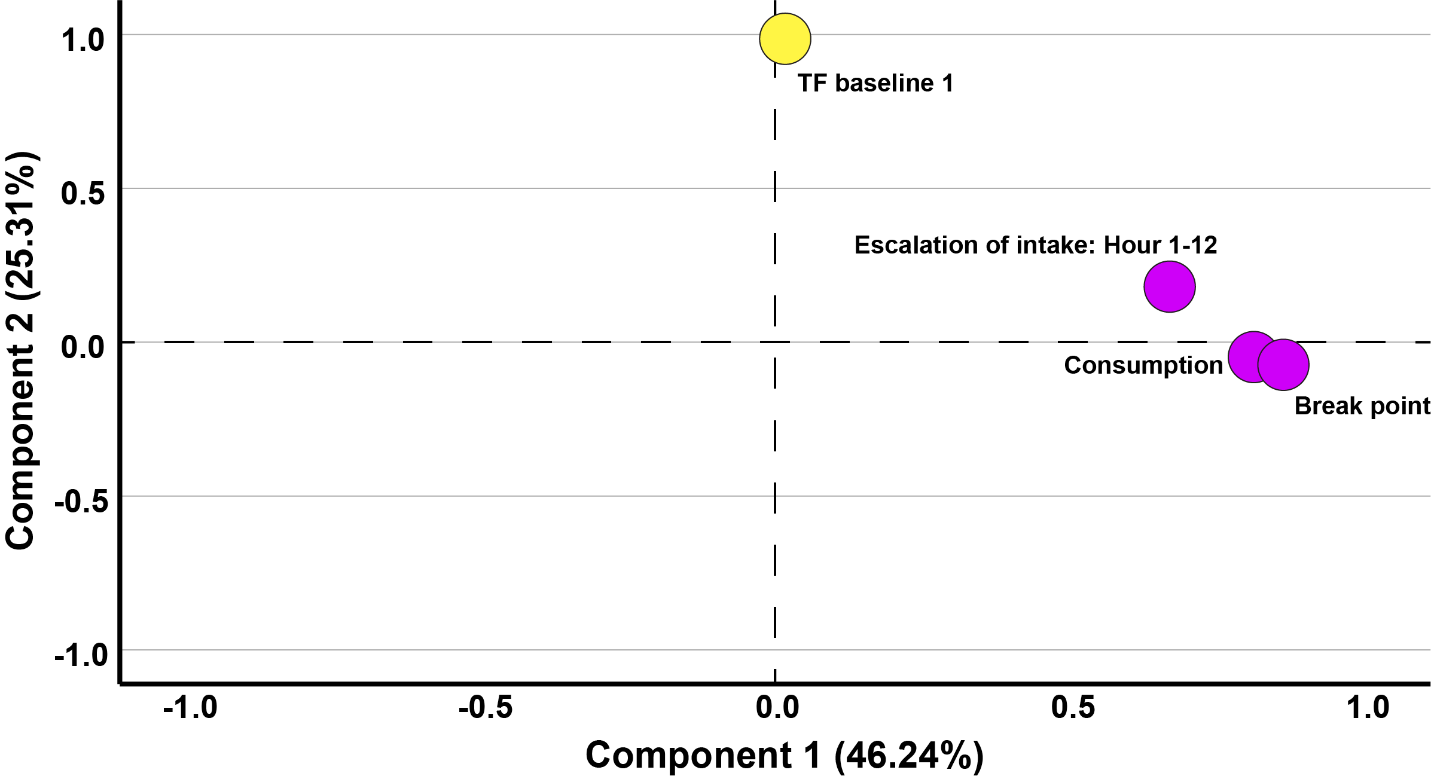


**Supplemental Figure 1** Principal component analysis for behavioral traits with significant QTLs. Two factors were identified and comprised 71.55% of total variance in the data. Heroin-taking behaviors accounted for 46.24% of variance (component 1, purple) with traits exhibiting strong loading (Break point: 0.86; Consumption: 0.81; and Escalation of intake: Hour 1-12: 0.67), and was mutually exclusive from baseline nociception prior to heroin experience (component 2, yellow) which accounted for 25.31% of variance with strong loading (0.99). Axes represent factor loading values for each trait within respective component. (n=874)

**
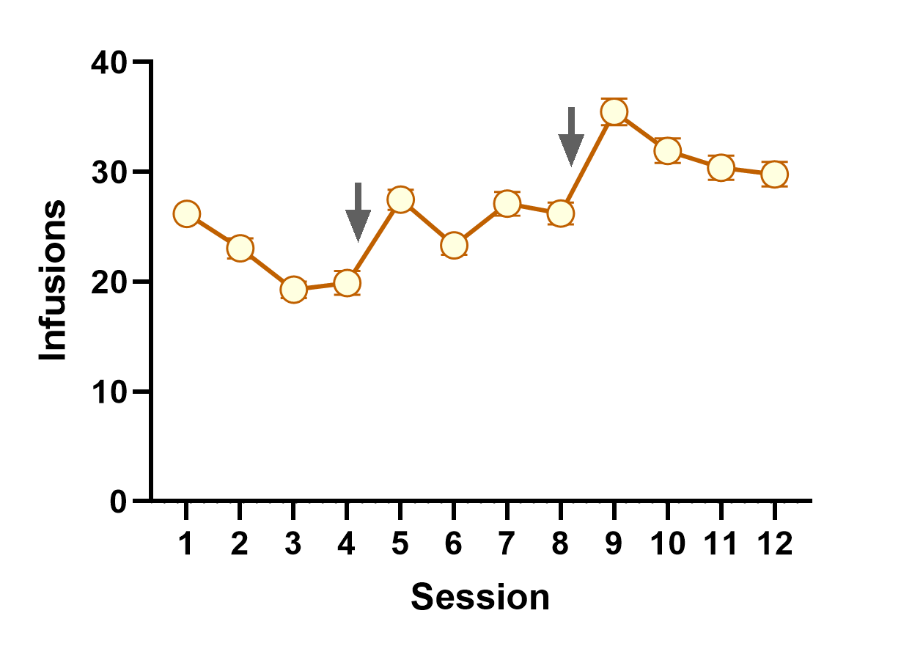
Supplemental Figure 2** Total number of heroin infusions earned across training sessions. Mean ± SEM for total heroin infusions (20 µg/kg/100 µl infusion) earned during heroin self-administration across training sessions. Animals underwent four training sessions a week with one day random day off between Monday-Friday. Grey arrows indicate brief periods of forced abstinence between training weeks. Rats increased heroin intake over the course of training (mixed effects ANOVA; F(5.90,5399)=43.24, p<0.001). (n=874)
