# Supplementary material for: Genome-wide association study reveals multiple loci for nociception and opioid consumption behaviors associated with heroin vulnerability in outbred rats
Source: Mol Psychiatry. 2025 Feb 25;30(8):3363–75. doi: 10.1038/s41380-025-02922-4 (PMC12240846; doi:10.1038/s41380-025-02922-4)
Supplement: Supplementary file 3 — GWAS dataset [file 41380_2025_2922_MOESM3_ESM.zip › gwas_report_u01_peter_kalivas_round10.2.1_threshold5.3591_n874_date2024-01-26_gwasversion_v0.2.0-11-g990844d.html]

Panel


GWAS REPORT
